# A Broad Spectrum Antiparasitic Activity of Organotin (IV) Derivatives and Its Untargeted Proteomic Profiling Using *Leishmania donovani*

**DOI:** 10.3390/pathogens11121424

**Published:** 2022-11-26

**Authors:** Obaid Hayat, Nazif Ullah, Muhammad Sirajuddin, Miriam A. Giardini, Jennifer V. Nguyen, Karol R. Francisco, Lawrence J. Liu, Yujie Uli Sun, Svetlana Maurya, Dominic McGrosso, David J. Gonzalez, Conor R. Caffrey, Anjan Debnath, Jair L. Siqueira-Neto

**Affiliations:** 1 Department of Biotechnology, Faculty of Chemical and Life Sciences, Abdul Wali Khan University, Mardan 23200, Pakistan; 2 Department of Chemistry, University of Science and Technology, Bannu 28100, Pakistan; 3 Center for Discovery and Innovation in Parasitic Diseases, Skaggs School of Pharmacy and Pharmaceutical Sciences, University of California San Diego, La Jolla, CA 92093, USA; 4 Department of Pharmacology, University of California San Diego, La Jolla, CA 92093, USA

**Keywords:** organotin (IV) compounds, antiparasitic activity, neglected tropical diseases, drug discovery

## Abstract

Metals have been used in medicine since ancient times for the treatment of different ailments with various elements such as iron, gold and arsenic. Metal complexes have also been reported to show antibiotic and antiparasitic activity. In this context, we tested the antiparasitic potential of 10 organotin (IV) derivatives from 4-(4-methoxyphenylamino)-4 oxobutanoic acid (MS26) against seven eukaryotic pathogens of medical importance: *Leishmania donovani*, *Trypanosoma cruzi*, *Trypanosoma brucei*, *Entamoeba histolytica*, *Giardia lamblia*, *Naegleria fowleri* and *Schistosoma mansoni*. Among the compounds with and without antiparasitic activity, compound MS26Et3 stood out with a 50% effective concentration (EC_50_) of 0.21 and 0.19 µM against promastigotes and intracellular amastigotes of *L. donovani,* respectively, 0.24 µM against intracellular amastigotes of *T. cruzi*, 0.09 µM against *T. brucei,* 1.4 µM against *N. fowleri* and impaired adult *S. mansoni* viability at 1.25 µM. In terms of host/pathogen selectivity, MS26Et3 demonstrated relatively mild cytotoxicity toward host cells with a 50% viability concentration of 4.87 µM against B10R cells (mouse monocyte cell line), 2.79 µM against C2C12 cells (mouse myoblast cell line) and 1.24 µM against HEK923 cells (human embryonic kidney cell line). The selectivity index supports this molecule as a therapeutic starting point for a broad spectrum antiparasitic alternative. Proteomic analysis of host cells infected with *L. donovani* after exposure to MS26Et3 showed a reduced expression of Rab7, which may affect the fusion of the endosome with the lysosome, and, consequently, impairing the differentiation of *L. donovani* to the amastigote form. Future studies to investigate the molecular target(s) and mechanism of action of MS26Et3 will support its chemical optimization.

## 1. Introduction

Metals have been used since ancient times in medicine with various elements such as arsenic, gold, and iron used to treat various ailments [1]. Metal elements are important in living systems making them appropriate and sometimes essential biological modulators. Iron is a key component of hemoglobin that transports oxygen [2]. Zinc, manganese and copper are essential components of several proteins and enzymes, including metalloproteases, copper cytochrome c oxidase, super oxidase and lysyl oxidase [3]. The antibiotic activity of metal-based organic molecules has also been explored. Metallic-based pharmaceuticals are used to treat fungal and bacterial infections, and cancer [4,5]. Metals oxidize by losing electrons and form positively charged ions that are soluble in biological fluids. In their cationic forms, metals fulfill their biological roles, often by electrostatic interactions with negatively charged molecules such as DNA and some amino acid side chains [6]. Despite having important roles in the homeostasis of complex organisms, metal complexes can also modulate biological activities and hence be explored as therapeutic agents.

Metal based compounds have been evaluated as experimental therapeutics against pathogens causing Neglected Tropical Diseases (NTDs). Platinum and palladium, for example, were active against *Trypanosoma cruzi* and inhibited the parasite’s growth through a dual mechanism of action involving an interaction with DNA and the production of toxic free radicals by bioreduction [7]. Palladium complexes were more active than metronidazole (the current drug treatment) against *Entamoeba histolytica*, however, the exact mechanism of action is not understood [8]. Rhodium (III) cationic complexes containing 2-hydroxybenzothiazole inhibit the growth of *Leishmania donovani* [9]. Further, ultra-structural analysis using electron microscopy suggested that the mitochondrion complex in *Leishmania* amastigotes is the primary site of action of the rhodium complex [10]. Auranofin, an oral gold-containing drug used for the treatment of rheumatoid arthritis, is effective against multiple parasites including *E. histolytica*, *Giardia lamblia*, *Naegleria fowleri*, *Cryptosporidium parvum*, *Trichomonas vaginalis*, *L. donovani*, *Toxoplasma gondii*, *T. cruzi*, *Schistosoma mansoni, Onchocerca* sp., *Brugia* sp. and *Taenia* [11,12,13,14,15,16,17,18,19,20]. Auranofin’s anti-inflammatory and redox effects make the drug a promising candidate for the treatment of parasitic infections, yet the its molecular mechanism of action remains unknown [21].

Organotin (IV) compounds have been widely investigated due to their biocidal activities against fungal and bacterial infections and cancer [22]. Tin (Sn) has played a vital role in advancement of organometallic chemistry, which started in 1949. Organotin carboxylates are used for chemical catalysis, synthesis, material chemistry, medicine and agriculture [23]. Organotin (IV)-based peptide derivatives were reported as active against *Leishmania tropica* (KWH23 strain) with IC_50_ values ranging from 0.4–2.8 µM and a selectivity index >500 for promastigotes and axenic amastigotes compared to human macrophages [24]. However, the mechanism of action of these compounds are still unknown.

Here, we investigated the anti-parasitic effects of 10 Organotin (IV) compounds derived from 4-(4-methoxyphenylamino)-4 oxobutanoic acid containing Sn, as well as the parent compounds (without Sn) in their acid and salt forms. The in vitro activity of the compounds was measured against seven eukaryotic pathogens that cause NTDs, namely *L. donovani*, *T. cruzi*, *T. brucei (Trypanosoma brucei)*, *E. histolytica*, *G. lamblia*, *N. fowleri* and *S. mansoni*. In attempt to elucidate the possible mode of action of the selective organotin (IV) compound. We also performed an untargeted proteomic analysis using *L. donovani* system and MS based proteomic approach for the identification of large number of proteins.

## 2. Material and Methods

### 2.1. Test Compounds and Reference Drug Controls Preparation

**Figure 1:**
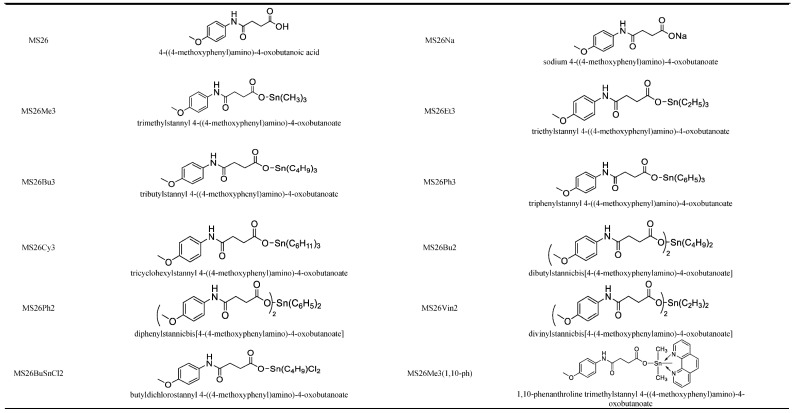
Chemical structure and IUPAC names of the organotin (IV) compounds.

A total of 12 compounds, including the ligand, 4-(4-methoxyphenylamino)-4 oxobutanoic acid (MS26), its sodium salt, sodium 4-(4-methoxyphenylamino)-4 oxobutanoate (MS26Na) and 10 organotin (IV) complexes derived from MS26Na were synthesized (Figure 1) at the Department of Chemistry, University of Science and Technology Bannu, Pakistan. Their detailed synthesis has been reported [25] The compounds were prepared as 20 mM stock solutions in DMSO. In addition, we used reference drugs as positive controls for each parasite, as follows: *L. donovani and N. fowleri* amphotericin B (Sigma A9528); *T. cruzi,* benznidazole (Sigma 419656) and K11777 (custom synthesis); *T. brucei,* pentamidine (Sigma P0547); *E. histolytica* and *G. lamblia*, metronidazole (Sigma M3761); and *S. mansoni*, praziquantel (Sigma P4668). We used 0.5% DMSO as a negative control for all assays and cevipabulin as a positive control for HEK293.

### 2.2. Leishmania Donovani Assays

Promastigotes of *L. donovani* from late log phase culture were diluted to 2.0 × 10^6^/mL in M-199 containing 10% FBS, 25 mM HEPES (Gibco 15630-080), 100 µM adenine, 12 mM NaHCO_3_, 10 µM folic acid, 1% penicillin/streptomycin (Gibco 15140-122) as described previously [26]. Compounds and controls were transferred from compound plates to 384 well assay plates with the use of an acoustic transfer system (ATS, EDC Biosystems, Fremont, CA, USA). Promastigotes were incubated with compounds at 29 °C for 48 h. To measure parasite viability, resazurin sodium salt (7-hydroxy-3H-phenoxazin3-one 10-oxide) was dissolved in PBS and added to each well at a volume of 5 µL (final concentration of 44 µM). After incubating for 4 h at room temperature, fluorescence (ex 571 nm/em 584 nm) was recorded with an Envision (Perkin Elmer, Waltham, MA, USA) microplate fluorescence/luminescence reader. To measure the EC_50_ value (the concentration at which parasite growth is inhibited by 50%), compounds were serially diluted 1000-fold in DMSO, with final assay concentrations ranging from 20 µM to 0.02 µM and tested in biological triplicates. For positive and negative control amphotericin B and 0.5% DMSO was used, respectively. Dose response and EC_50_ analyses were obtained using GraphPad Prism 9 Software (Graph Pad Software Inc., San Diego, CA, USA).

For the intracellular amastigote stage assay, *L. donovani* was incubated at a density of 1 × 10^6^ parasites/mL for six days at 29 °C before infection to enrich the proportion of metacyclic promastigotes. B10R monocyte cell lines were cultured in RPMI supplemented with 10% FBS and 1%penicillin/streptomycin at 37 °C in 5% CO_2_. After 72 h, cells were counted with a hemocytometer and mixed with the six-day-old parasites. Into 384-well assay plates, that had been pre-spotted with compounds diluted 1000-fold in DMSO, with final assay concentrations ranging from 20 µM to 0.02 µM and tested in biological triplicates., 500 B10R cells and 7000 parasites were dispensed per well in a final volume of 50 µL using a Multidrop Combi liquid dispenser (ThermoScientific, Waltham, MA, USA). Plates were incubated at 37 °C and 5% CO_2_ for 72 h. Cultures were then fixed with 4% formaldehyde solution for 30 min and stained with 0.5 µg/mL DAPI (diamidino-2-phenylindole). Plates were then imaged using an ImageXpress Micro XLS automated high content imager (Molecular Devices, San Jose, CA, USA) using a 10× air objective. A custom image analysis module generated in ImageXpress was used to count the nuclei of host and parasite cells. EC_50_ values were obtained using the GraphPad Prism 9 Software with biological triplicates.

### 2.3. Trypanosoma cruzi Assay

Mouse C2C12 myoblasts (ATCC CRL-1772) were cultivated in RPMI 1640 medium supplemented with 5% FBS and kept at 37 °C and 5% CO_2._ *T. cruzi* CAI-72 trypomastigotes were obtained from the supernatant of C2C12 cells infected four to seven days previously [27]. For the assay, 500 C2C12 cells were mixed with 7500 *T. cruzi* parasites and seeded into 384-well plates at a 50 µL final volume. Test compounds and reference drugs (Benznidazole and K11777) were pre-spotted into the 384 well black bottom plates prior to the addition of cells and parasites with final assay concentration of 20 µM to 0.02 µM. The plates were incubated at 37 °C^,^ 5% CO_2_ for 72 h. Cultures were then fixed with 4% formaldehyde and 0.5 µg/mL DAPI (diamidino-2-phenylindole) was added for staining nucleic acids. Plates were kept protected from light for 1 h and then imaged in an ImageXpress Micro XLS automated high content imager (Molecular Devices, San Jose, CA, USA). Imaging and data analysis were performed as described for *L. donovani* amastigotes on biological triplicates.

### 2.4. Trypanosoma brucei Assay

Bloodstream forms of *T. b. brucei* Lister 427 were cultured in complete HMI-9 medium [28] in T25 suspension culture flasks (Thermo Scientific, Cat. 12-566-84) and maintained at 37 °C and 5% CO_2_. Trypanosomes were kept in log-phase growth and passaged every 48 h. Test compounds were serially diluted in DMSO and added to 96-well plates to give final assay concentrations ranging from 4 µM to 0.87 nM (1 µL; 0.5% total DMSO). Pentamidine were used as a positive control for *T. brucei* assay. Compounds were diluted with fresh HMI-9 medium (99 µL/well). Parasites in log-phase were suspended at 2 × 10^5^ parasites/mL in HMI-9 medium and then added to each well (100 µL) to give 2 × 10^4^ trypanosomes/well. Assay plates were incubated at 37 °C and 5% CO_2_ for 72 h. Parasite lysate was obtained by addition of 50 µL/well of lysis solution (0.012% saponin, 0.12% Triton X-100, 7.5 mM ETDA, 30 mM Tris, pH 7.5) containing 0.3 µL/mL SYBR Green I (10,000× in DMSO; Invitrogen, Carlsbad, CA, USA). Assay plates were incubated in the dark for 1 h at room temperature. Fluorescence was measured at 485 nm excitation and 535 nm emission wavelengths using an EnVision multilabel plate reader (PerkinElmer, Waltham, MA, USA). In each assay plate, parasite viability was normalized relative to positive (pentamidine) and negative (0.5% DMSO) controls in each assay plate. Assays were performed in biological and technical duplicates, and dose–response and EC_50_ data were generated with GraphPad Prism 9 using a sigmoidal four parameter logistic curve.

### 2.5. Entamoeba histolytica Assay

Trophozoites of *E. histolytica* HM1:IMSS were maintained in vitro as described [29] in TYI-S-33 medium supplemented with penicillin (100 U/mL) and streptomycin (100 µg/mL). Test compounds were serially diluted and transferred to a white solid bottomed 96-well tissue culture plate (E&K Scientific, Swedesboro, NJ, USA) followed by addition of 5000 *E. histolytica* trophozoites per well for a final volume of 100 µL TYI-S-33 medium and a final concentration of compounds ranging from 50 to 0.39 µM. Metronidazole were used as positive control and assay plates were incubated for 48 h at 37 °C. The plates maintained kept anaerobically using the GasPak EZ Anaerobic Gas Generating Pouch System (VWR). Parasite viability was measured in triplicate using the CellTiter-Glo luminescence kit (Promega G9243) in an Envision plate reader (Perkin Elmer, Waltham, MA, USA) [29]. EC_50_ values were obtained using GraphPad Prism 9 Software.

### 2.6. Giardia lamblia Assay

Trophozoites of *G. lamblia* (WB strain) were maintained in vitro as described [30] in TYI-S-33 medium supplemented with penicillin (100 U/mL) and streptomycin (100 µg/mL). Cells were harvested during the logarithmic phase of the growth for antiparasitic assessment [31]. Test compounds were serially diluted and transferred to a white solid bottomed 96-well tissue culture plate (E&K Scientific, Swedesboro, NJ, USA) followed by addition of 5000 *G. lamblia* trophozoites for a final volume of 100 µL of TYI-S-33 medium and a final concentration of compounds ranging from 50.0 to 0.39 µM. Metronidazole was used as a positive control and assay plates were incubated for 48 h at 37 °C in the GasPak EZ Anaerobic Gas Generating Pouch System (VWR). Parasite viability was measured in triplicate using the CellTiter-Glo luminescence kit in an Envision plate reader [30]. EC_50_ values were obtained using the GraphPad Prism 9 Software.

### 2.7. Naegleria fowleri Assay

*N. fowleri* (KUL strain) was cultured in Nelson’s medium supplemented with 10% FBS and kept at 37 °C. For all experiments, trophozoites were harvested during the logarithmic phase of growth [32]. Test compounds were serially diluted and transferred to a white solid bottomed 96-well tissue culture plate (E&K Scientific, Swedesboro, NJ, USA) followed by addition of 10,000 *N. fowleri* trophozoites in Nelson’s medium in a final volume of 100 µL and a final concentration of compounds ranging from 50 to 0.39 µM. Amphotericin B was used as positive control and assay plates were incubated for 48 h at 37 °C. Parasite viability was measured as described for *E. histolytica* and *G. lamblia* [33]. EC_50_ values were obtained using the GraphPad Prism 9 Software.

### 2.8. Schistosoma mansoni Assay

The Naval Medical Research Institute (NMRI) isolate of *S. mansoni* was used. The parasite was cycled between Biomphalaria glabrata snails and male Golden Syrian hamsters (infected at 4–6 weeks of age) as intermediate and definitive hosts, respectively. Hamsters were infected and maintained in accordance with protocols approved by the Institutional Animal Care and Use Committee (IACUC) at the University of California San Diego. The acquisition, preparation and in vitro maintenance of *S. mansoni* have been described [34,35]. Briefly, adult worms were harvested from hamsters 42 days post-infection in RPMI or DMEM, and washed five times prior to maintenance overnight at 37 °C and 5% CO_2_ in Basch medium [36] containing 4% heat-inactivated FBS, 100 µg/mL streptomycin and 100 U/mL penicillin.

Ex vivo screens with adults employed 24-well flat-bottomed plates and approximately 5 males and 2–3 females/well. The complex and often dynamic phenotypic responses that the schistosome parasite is capable of were observed using a Zeiss Axiovert A1 inverted microscope at 24 h. Observations were classified using a constrained nomenclature that involves simple “descriptors” to convey changes in shape, motility and appearance, and the inability of the male worm to adhere to the bottom of the well, compared to DMSO controls [35]. Each descriptor was given a value of 1 and these were added up to yield a “severity score” with a maximum of 4. Evidence of degeneracy or death was awarded a value of 4 as was damage to the tegument (parasite’s surface) [37,38,39,40] on the understanding this would compromise survival in vivo. Data are reported as the least concentration across four concentrations tested (10, 5, 2.5 and 1.25 µM) that yielded observable changes in the parasite. Data were averaged across two experiments for each compound concentration tested.

### 2.9. HEK293 Assay

HEK-293T cells were cultured in DMEM (Gibco) supplemented with 10% heat inactivated FBS (Corning) and 1% penicillin-streptomycin (Gibco). Cells were maintained in vented T75 cell culture flasks (Thermo Scientific, Cat. 12-565-349) at 37 °C and 5% CO_2_, and were passaged every 72 h. Test compounds were serially diluted in DMSO and added to 96-well plates to give final assay concentrations ranging from 4 µM to 0.87 nM (1 µL; 1% total DMSO). Compounds were diluted with fresh DMEM (49 µL/well). HEK-293 cells were suspended at 4 × 10^5^ cells/mL in DMEM and added to each well (50 µL/well) to give 2 × 10^4^ cells/well. Assay plates were incubated at 37 °C and 5% CO_2_ for 48 h, followed by addition of 20 µL of 0.5 mM resazurin (Alfa Aesar, Cat. B21187) in PBS to each well. Plates were incubated in the dark for 4 h at 37 °C, and fluorescence was measured at 531 nm excitation and 595 nm emission wavelengths using an EnVision multilabel plate reader. In each assay plate, cell viability was normalized relative to positive and negative controls. Assays were performed in biological and technical duplicates, and dose–response and CC_50_ data were generated with GraphPad Prism 5 using a sigmoidal four parameter logistic curve.

### 2.10. Proteomic Analysis

#### 2.10.1. Protein Preparation

B10R culture cells (infected or uninfected with *Leishmanaia donovani*) were collected and washed twice in cold PBS. The cell pellets (~10^6^ cells/pellet) were snap-frozen and stored at −80 °C. To extract the proteins, pellets were thawed, and the cells were resuspended in 200 µL buffer containing 6 M urea, 7% SDS, 50 mM TEAB, protease inhibitor cocktail (Roche, cat. no. A32963), phosphatase inhibitor (Roche, cat. no. A32957), with pH adjusted to 8.1 with phosphoric acid. The cells were then lysed using a sonicating probe 200 µL. The samples were reduced in 500 mM DTT at 47 °C for 30 min and alkylated in 500 mM IAA in the absence of light, at room temperature for 45 min, followed by quenching in 500 mM DTT for 5 min. The samples were then acidified with 12% phosphoric acid and mixed in a 7:1 ratio with binding buffer containing 90% methanol, 50 mM TEAB, pH 7.1, adjusted with phosphoric acid and loaded on to S-Trap columns (Protifi). Column digestion occurred with the addition of 5 µg mass spectrometry grade trypsin in 50 mM TEAB for 3 h at 47 °C. Peptides were eluted first in 50 mM TEAB, then 5% formic acid, and finally 50% acetonitrile 5% formic acid, and completely dried using a speed vacuum concentrator. Samples were then desalted with column C18 Sep-Paks (Waters) and eluted with 40% and 80% acetonitrile containing 0.5% acetic acid. The concentration of desalted peptides was determined and 50 µg aliquots of each sample were dried in a speed vacuum concentrator. Ten micrograms of each sample was also pooled for bridge channels (depending on experiment and as listed in the Supplementary Files) as previously described [41]. Mass offsets for each TMT set were accounted for in the database searches according to the manufacturer’s report per lot number. The lot number for TMT reagents was VL312003. Each sample or bridge channel was resuspended in 30% dry acetonitrile in 200 mM HEPES (pH 8.5) for TMT labelling with 7 μL of the appropriate TMT reagent, as previously described [42]. Reagent131 (ThermoFisher, Waltham, MA, USA) was used to bridge between MS runs. Remaining reagents were used to label samples in random order as indicated in the Supplementary Files. Labelling was performed at room temperature for 1 hand quenched with 8 μL 5% hydroxylamine (Sigma). Labelled samples were acidified by adding 50 μL 1% TFA. After TMT labelling, each 10-plex experiment was combined, desalted (C18 Sep-Paks) and dried in a speed vacuum concentrator.

#### 2.10.2. LC-LC-MS^n^ Proteomics

Basic pH reverse-phase LC and data acquisition through LC–MS2/MS3 were performed as previously described [41]. Briefly, 75 min linear gradients of acetonitrile were passed on C18 columns using an Ultimate 3000 (ThermoFisher, Waltham, MA, USA) high-performance liquid chromatography (HPLC) system with a fraction collector, degasser, and variable-wavelength detector. Separation was performed using a C18 column (ThermoFisher, Waltham, MA, USA, 4.6 mm by 250 mm) on a 22% to 35% 75 min gradient of acetonitrile and 10 mM ammonium bicarbonate (Fisher) at 0.5 mL/min. The resulting 96 fractions were combined as previously described [43]. Fractions were dried under vacuum. Fractions were then analyzed using tandem mass spectrometry (MS2/MS3) on an Orbitrap Fusion mass spectrometer (ThermoFisher, Waltham, MA, USA) with an in-line EASY-nLC 1000 instrument (ThermoFisher, Waltham, MA, USA). Separation and acquisition settings were performed using previously defined methods [44].

#### 2.10.3. Proteomic Data Analysis

Mass spectrometry data were searched using Proteome Discoverer 2.5 software. Data were searched against the reference proteome for *Mus musculus* downloaded from Uniprot.com on 1/30/2021. The SEQUEST search algorithm was employed to align MS2 spectral data against theoretical peptides generated in silico [45]. Precursor tolerance was set to 10 ppm and fragment tolerance was set to 0.6 Da. Static modifications were specified for TMT labels on N-termini and lysine residues, as well as for carbamidomethylation of cysteines. Dynamic modifications were set for oxidation of methionine. A 1% false discovery rate was specified for the decoy database search [46]. Peptide spectral match abundances were summed to the protein level and resultant summed values were normalized against the average value for each protein divided by the median of all average protein values. A second normalization step was performed whereby the abundance value for each protein per sample was divided by the median value for each channel which had itself been divided by the overall dataset median. Differentially abundant proteins were identified using a π score, a significance metric that incorporates both fold changes and traditional *p*-value based significance scores, determined through a Student’s *t*-test with or without Welch’s correction [47]. Gene ontology and molecular networks were created using Enrichr and StringDB, respectively [48,49,50].

## 3. Results

### 3.1. Compounds Screening against Different Parasites and Cell Lines

The selected Organotin (IV) compounds (MS26BuSnCl2, MS26Bu2, MS26Et3, MS26Bu3, MS26Oct2, MS26Me3, MS26Ph3, MS26Cy3, MS26Vin2 and MS26(1,10-ph), and their parent compounds without tin (MS26 and MS26Na) were tested against seven NTD-causing parasites. The structures are presented in Figure 1 on page 3. The antiparasitic activity and/or EC_50_ values, and cytotoxicity (CC_50_) values are reported in Table 1; also, the data specifically related to the antileishmanial activity and mechanism of action are presented in the Supporting Information (Figures S1–S3).

The parent compounds MS26 and its sodium salt version MS26Na showed mild activity against the trypanosomatids *L. donovani* and *T. cruzi*, with EC_50_ values ranging from 1.08 to 4.27 µM against the intracellular stages of both parasites and were inactive against all other parasites up to the maximum concentrations tested. Compounds including Sn in the carboxylic acid position showed varied antiparasitic activities compared to the parent compounds. Some compounds such as MS26Oct2, MS26Vin2, MS26(1,10-Ph) and MS26BuSnCl2, were in active against all parasites, and were not significantly toxic to the mammalian cells either, making the compounds apparently inert in the tested biological systems. The difference in activity of the various triorganotin (IV) carboxylates could be due to the difference in lability of the R-Sn bond, i.e., the difference in electron-withdrawing and electron-donating character of the R moiety. The activity of the organotin (IV) carboxylate compounds is high due to the fact that they exist in polymeric form [25], and in the solution state only slowly lose the active part to the target.

Among the tested compounds, MS26Et3 and MS26Bu2 were active against *L. donovani* promastigotes (insect stage) with EC_50_ values of 0.21 and 0.76 µM, respectively (Table 1; dose response curves presented in Supporting Information Figure S1). Against intracellular amastigotes (stage present in the mammalian host), the EC_50_ values were 0.19 and 0.28 µM, respectively, (Table 1; dose response curve and image-based analysis presented in Supporting Information Figures S2 and S3). Against the host B10R monocytes, the respective CC_50_ values were 2.41 and 1.06 µM (Table 1; dose response curve in Supporting Information Figure S3). Thus, the selectivity indices (SI) for MS26Et3 and MS26Bu2 calculated between the intracellular amastigotes and the host cells were 13.1 and 3.78, respectively. MS26Et3 was also active against *T. cruzi* intracellular amastigotes with an EC_50_ value of 0.24 µM and a CC_50_ value of 2.79 µM against C2C12 cell lines (SI = 11.7; Table 1).

Out of 12 compounds, five, including MS26Et3, MS26Bu3, MS26Ph3, MS26Cy3 and MS26Bu2, were active against *T. brucei* at the tested concentrations, with EC_50_ values ranging from 0.05–0.29 µM. Interestingly, three of these compounds, MS26Et3, MS26Bu3 and MS26Bu2, were also active against both forms of *L. donovani* and against *T. cruzi.* Additionally, MS26Ph3 was active against both forms of *L. donovani* whereas MS26Cy3 was active against *T. brucei.* Compounds that were active against *T. brucei* were also found to have some cytotoxicity against the host cell lines (CC_50_ values ranging from 0.11–0.56 µM), however, SI values were >1.0. The selected compounds were also screened against *E. histolytica, G. lamblia* and *N. fowleri.* MS26Et3 and MS26Bu3 displayed anti-parasitic activity against *N. fowleri* with EC_50_ values of 1.4 µM and 1.5 µM, respectively. The other active compounds were MS26Ph3 (EC_50_ of 3.2 µM) and MS26Cy3 (EC_50_ of 7.5 µM). The remaining compounds were less active at the maximum concentration of 10 µM against *N. fowleri*. Only three compounds MS26Et3, MS26Bu3 and MS26Ph3 were active against *G. lamblia* with EC_50_ values of 8, 1.03 and 5.03 µM, respectively. MS26Et3 and MS26Bu3 were active against *E. histolytica* with EC_50_ values of 5.6 and 8.7 µM, respectively.

Compounds were tested for bioactivity against adult *S. mansoni* at 10, 5, 2.5 and 1.25 µM for 24 h using an observational analysis and a scoring system that considers the many changes that this flatworm can manifest in response to chemical stimuli. MS26, MS26Na, MS26Vin2, MS26(1,10-Ph) and MS26BuSnCl2 were inactive (severity score of zero out of a maximum of 4) at 10 µM. MS26Oct2 was partially active at 10 µM yielding a severity score of 2, whereby the worms exhibited an uncoordinated motility and an inability to adhere to the floor of the well with either the oral or ventral sucker. MS26Me3 was lethal at 10 µM and at 5 µM yielded the same response as MS26Oct2 at 10 µM. In contrast, the five remaining compounds, MS26Et3, MS26Bu3, MS26Ph3, MS26Cy3 and MS26Bu2, at all concentrations, caused lethal degenerative changes in the parasite, including damage to the surface tegument, with severity scores of 4.

### 3.2. Proteomic Analysis

**Figure 2:**
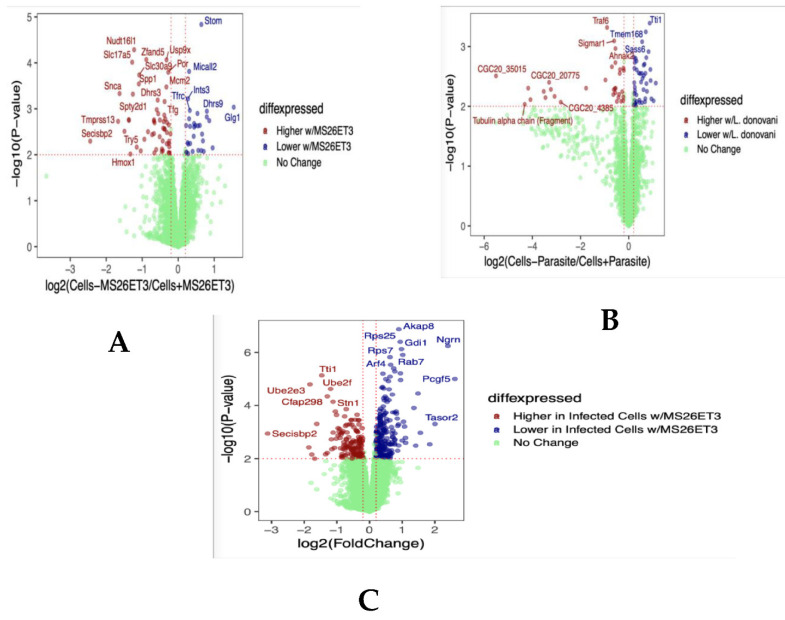
Untargeted proteomic analysis with MS26Et3 (**A**) Differential expression of proteins in B10R cells treated and untreated with MS26Et3. (**B**) Differentially expressed proteins in B10R cells infected and not infected with *L. donovani*. (**C**) Differentially expressed proteins in *L. donovani*-infected B10R cells treated and untreated with MS26Et3.

To gain insight into the possible the mechanism of action of the active organotin compound, MS26Et3, in the *Leishmania donovani* system, we performed untargeted proteomic analysis under different conditions. First, we compared the protein expression profile in non-infected B10R cells in the presence and absence of MS26Et3. That first experiment provided the overall changes in host protein abundance when the compound was added to the cell, indicating how the compound modulated host proteome (Figure 2A). We also analyzed the protein expression profile in B10R cells that had been infected with *L. donovani* but without compound treatment, providing information related to how the parasite infection changes the host protein abundance (Figure 2B). For these first two experiments, we saw dozens of proteins with significant change in abundance comparing the scenarios. Finally, we compared the expression profiles in infected B10R cells in the presence and absence of exposure to MS26Et3 (Figure 2C).

We observed that 238 host proteins were decreased in abundance following the introduction of MS26Et3 to infected cells. We decided to focus on the parasite proteins. Of the >200 parasite proteins quantified, only Rab7 was found to be differentially expressed. Rab7 is a small GTPase mainly associated with the formation of endosomes [51]. Recruitment of Rab7 to phagosomes is essential for their fusion with late endosomes or lysosomes [52]. Lack of recruitment could indicate the poor fusing with endocytic organelles. Without phagolysosome formation, the differentiation from promastigote to amastigote could be impaired—a possible mechanism of antiparasitic activity [53], however, this hypothesis must be experimentally tested.

## 4. Discussion

The development of a single drug with broad spectrum anti parasitic activity is an attractive goal in the context of Neglected Tropical Diseases which remain a major public health burden in developing countries and lack adequate therapeutic options [54]. Organotin (IV) and its complexes have several therapeutic applications, including anticancer, anti-inflammatory, antibacterial and antiparasitic activities [55,56,57,58]. In this study, 10 organotin (IV) compounds were screened against parasites that cause disease in humans: *L. donovani*, *T. cruzi*, *T. brucei*, *G. lamblia*, *E. histolytica*, *N. fowleri* and *S. mansoni*.

Of the organotin (IV) compounds, MS26Et3 at 5 µM eliminated >95% of *L. donovani* and *T. cruzi* intracellular parasites infecting a mammalian host cell and >99% of *T. brucei*. The activity against *L. donovani* intracellular amastigotes and promastigotes, with EC_50_ values = 0.21 and 0.19 µM, respectively, as compared to the positive control amphotericin B. An earlier study with organotin (IV) derivatives [24] reported IC_50_ values ranging from 0.01 µg/mL to 0.14 µg/mL against *L. tropica* promastigotes, and 0.1 µg/mL to 1.0 µg/mL against axenic amastigotes. The target of the compounds was speculated to be leishmanolysin (GP63) receptor. Organometallic complexes of triphenyl tin were also effective both in vitro and in vivo against *L. donovani* with an IC_50_ of 0.05 µg/mL [59].

In the search for antichagasic drugs, metal complexes represent an alternative chemical space and have been explored [7]. Here, we found that MS26Et3 was effective against *T. cruzi* amastigotes with an EC_50_ value of 0.24 µM which compares well with a CC_50_ value of 2.79 µM for the C2C12 host cells. Against *T. brucei,* MS26Et3 also effectively inhibited parasite growth with an EC_50_ 0.09 µM a value that compares well with data from a previous study demonstrating the potency of diorganotin against *T. b. brucei*, *T. b. gambiense* and *T. b. rhodesiense*, with EC_50_ values in the range of 0.15 to 0.75 µM.

Several compounds of diorganotin complexes were evaluated against *Candida albicans* and no changes were found in DNA integrity and mitochondrial functions, but a reduction in the ergosterol biosynthesis was observed [60], indicating that the target could be related to this pathway. The MS26Et3 showed antiparasitic activity against all three kinetoplastids with sub-micromolar IC_50_s, and against *N. fowleri* with an IC_50_ of 1.40 µM. The ergosterol biosynthesis precursor in kinetoplastids is lanosterol, while the de novo biosynthesis of ergosterols in amoebae occurs from cycloartenol which is a typical precursor in photosynthetic organisms for example algae and plants [61]. Ergosterol is also essential for the integrity of cell membrane in *Naegleria* sp. [62]. In vitro activity of the compounds in nano to micromolar ranges warrant the subsequent evaluation for in vitro toxicity [63]. Our observed IC_50_ were all in the lower micromolar or sub-micromolar ranges against all tested parasites. The antiparasitic activity of the Organotin (IV) compounds has been reported to be associated with disruption of microtubules [64,65].

Metal complexes, especially copper(II) complexes previously showed significant anti-schistosomal properties, inducing severe tegmental damage and a significant decrease in the production of eggs [66]. This suggests that metal-based compounds represent a viable source of novel anthelmintic drugs.

The schistosome tegumental membranes contribute to many processes at the host–parasite interface and numerous molecular pathways that are represented at the host–parasite boundary are anti-parasitic drug targets [67]. It has been reported that low concentrations of organometallic compounds (0.1–1 µM) affect metabolic processes in living human cells, whereas higher concentrations >10 µM change the physical and mechanical properties of cell membranes [68]. The nature and size of the R group attached to Sn (IV) affected the in vitro antiparasitic activity and with the increase in the percent CH of the R groups, decrease in activity [69].

The summation of these observations, therefore, suggests that these compounds are promising candidates against multiple parasites.

Mechanistic studies on organotin compounds are rare, but several compounds such as triphenyl tin salicylanilide [Ph3Sn(OSal·TSCZH)] (TTST) were evaluated against visceral leishmaniasis and found to inhibit superoxide dismutase, release the toxic superoxide radical and downregulate parasitic infection [59]. From our proteomic analysis, we concluded that 238 proteins decreased in abundance following the introduction of MS26Et3 to infected B10R cells. More than 200 parasite proteins were also detected, of which Rab7 was found to be decreased in abundance in infected cells that had been exposed to MS26Et3. Rab7 is a small GTPase primarily associated with late endosomes formation [51]. The recruitment of Rab7 to phagosomes is essential for their fusion with late endosomes and/or lysosomes [52].We hypothesize that a downstream effect of MS26Et3 is the decrease of Rab7 expression that then impairs the fusion of the phagosome with the lysosome, thereby jeopardizing the formation of the environment required by the parasite for its differentiation from promastigote to amastigote [53].Rab7 in its active form is detected on the phagosome membrane and associates with its only known effector protein, RILP (Rab7-interacting lysosomal protein), which in turn bridges phagosomes with dynein-dynactin, a microtubule-associated motor complex. This complex not only displaces phagosomes in the centripetal direction, but also promotes the extension of phagosome tubules toward the late endocytic compartments [52]. Several microorganisms avoid the recruitment of Rab7 to phagosomes to survive in their host cells; for example, *Toxoplasma gondii* is present within a vacuole that does not interact with endocytic organelles and also does not acquire Rab7 [70]. For mycobacterium, phagosomes can fuse with early endosomes but fail to fuse with lysosomes [71,72], resulting in the accumulation of the small GTP-binding protein, Rab5, and a failure to acquire Rab7 [73].The molecular target(s)of MS26Et3 and the mechanism by which Rab7 expression is altered are still unknown and should be investigated in the *L. donovani* system. Further, the mechanism of action of MS26Et3 against the other parasites herein investigated should be explored.

## 5. Conclusions

In the present study, a total of 12 compounds including the ligand, 4-(4-methoxyphenylamino)-4 oxobutanoic acid (MS26) and its salt version, sodium 4-(4-methoxyphenylamino)-4 oxobutanoate (Ms26Na) were evaluated for their antiparasitic activity against seven different parasites of medical importance and to investigate their possible mode of action against the parasite of *L. donovani* specifically. It is concluded that the compound MS26Et3 will act as effective anti-leishmanial and anti-parasitic agents. We can interpret from these data that the compound MS26Et3 was active against multiple parasites and Helminthes, showing minor cytotoxicity against host cells and limited the recruitment of Rab7 to prevent the infection in *L. donovani* system. Moreover, the compound MS26Et3 represents a starting point for the development of a broad-spectrum metal-based antiparasitic drug.

## Figures and Tables

**Table 1:** Bioactivity and cytotoxicity profiles of organotin (IV) derivatives against different parasites and mammalian cell lines. For *S. mansoni*, the data display the least concentration at which a severity score of 4 was recorded after 24 h. Background color: a range of color from green to red was set for each cell or parasite. Green refers to low cytotoxicity to host cells or potent antiparasitic activity; red refers to high cytotoxicity to host cells or weak/non-observed antiparasitic activity up to the highest tested concentration.

Compounds	MW	CC_50_ (_µM_)			EC_50_ (_µM_)								*SI Index*		
		C2C12	HEK923	B10R	*L. donovani* (pro)	*L. donovani* ntracelular amastigotes	*T. cruzi* (infecting C2C12)	*T. brucei*	*E. histolytica*	*G. lamblia*	*N. fowleri*	*S. mansoni*, adult worm	SI^a^	SI^b^	SI^C^
MS26	223.23	>20.0	>4.00	2.38	>20.0	1.08	3.21	>4.0	>10.0	>10.0	>10.0	>10.0	2.02	6.23	1
MS26Na	245.1	1.61	>4.00	4.55	0.17	3.88	4.27	>4.0	>10.0	>10.0	>10.0	>10.0	1.06	0.38	1
MS26Me3	386.03	9.41	1.24	4.87	>20.0	3.26	>20.0	>4.0	>10.0	>10.0	>10.0	10	1.45	0.47	0.31
MS26Et3	428.11	2.79	0.56	2.41	0.21	0.19	0.24	0.09	5.6	8	1.4	5	13.1	11.7	6.22
MS26Bu3	512.3	0.22	0.14	0.17	0.22	0.21	0.11	0.05	8.7	1.03	1.5	1.25	0.8	2	2.8
MS26Ph3	572.24	0.41	0.36	0.29	0.39	9.1	>20.0	0.09	>10.0	5.3	3.2	1.25	0.1	0.03	4
MS26Cy3	590.38	>20.0	0.11	0.11	>20.0	>20.0	0.3	0.08	>10.0	>10.0	7.5	1.25	0.01	66	1.37
MS26Bu2	677.4	0.14	0.37	1.06	0.76	0.28	0.53	0.29	>10.0	>10.0	>10.0	1.25	3.78	0.27	1.28
MS26Oct2	789.59	>20.0	>4.00	>20.0	>20.0	>20.0	>20.0	>4.0	>10.0	>10.0	>10.0	>10.0	1	1	1
MS26vin2	617.2	>20.0	>4.00	>20.0	>20.0	>20.0	>20.0	>4.0	>10.0	>10.0	>10.0	>10.0	1	1	1
MS26(1,10-Ph)	566.24	>20.0	>4.00	6.25	>20.0	>20.0	13.6	>4.0	>10.0	>10.0	>10.0	>10.0	1	1.48	1
MS26BuSnCl2	468.95	18.1	>4.00	0.62	>20.0	>20.0	>20.0	>4.0	>10.0	>10.0	>10.0	>10.0	0.031	0.91	1
AmpB											0.09				
Benznidazole							4.71								
Pentamidine								0.01							
Metronidazole									4.4	5.7					
Praziquantel												1.25			

**SI^a^**: Calculated ratio between CC_50_ in B10 R cells and EC_50_ against *L. donovani* intracellular amastigotes. **SI^b^**^:^ The ratio between CC_50_ in C2C12 cell lines and EC_50_ against *T. cruzi* intracellular amastigotes. **SI^c^**: The ratio calculated between CC_50_ in HEK923 cell lines and EC_50_ against *T. brucei.*

## Data Availability

The complete proteomic data was made available as supplemental material.

## Supplementary Materials

The following supporting information can be downloaded at: https://www.mdpi.com/article/10.3390/pathogens11121424/s1, Figure S1: Dose response curves of the potent organotin (IV) derivatives and amphotericin B (control drug) against the promastigotes of *L. donovani*; Figure S2: Inhibition of intracellular *L. donovani* amastigotes by organotin (IV) derivatives. ImageXpress Micro XLS automated microscope was used at 10× magnification for imaging host cells and parasites stained with DAPI; Figure S3: Dose-response curves of the potent organotin compounds and amphotericin B (reference drug) against intracellular amastigotes of *L. donovani*. Table S1: Proteomics Host cell with MS26Et3; Table S2: Proteomics Infected cells with MS26Et3; Table S3: Proteomics Leish infection.
