# Supplementary material for: A Broad Spectrum Antiparasitic Activity of Organotin (IV) Derivatives and Its Untargeted Proteomic Profiling Using Leishmania donovani
Source: Pathogens. 2022 Nov 26;11(12):1424. doi: 10.3390/pathogens11121424 (PMC9785441; doi:10.3390/pathogens11121424)
Supplement: Supplementary file 1 [file pathogens-11-01424-s001.zip › pathogens-2031974-supplementary.pdf]

A broad spectrum antiparasitic activity of Organotin (IV) derivatives and its untargeted proteomic profiling using *Leishmania donovani*

Obaid Hayat[1], Nazif Ullah[1], Muhammad Sirajuddin[2], Miriam A Giardini[3], Jennifer V. Nguyen[3], Karol R. Francisco[3], Lawrence J. Liu[3], Yujie Uli Sun[3], Svetlana Maurya[4], Dominic McGrosso[4], David J. Gonzalez[3,4], Conor R. Caffrey[3], Anjan Debnath[3], Jair L. Siqueira-Neto[3],

Affiliation

1 Department of Biotechnology, Faculty of Chemical and Life Sciences, Abdul Wali Khan University, Mardan, Pakistan

2 Department of Chemistry, University of Science and Technology, Bannu, Pakistan

3 Center for Discovery and Innovation in Parasitic Diseases, Skaggs School of Pharmacy and Pharmaceutical Sciences, University of California San Diego, La Jolla, CA, USA

4Department of Pharmacology, University of California San Diego, La Jolla, CA, USA

Corresponding author: jairlage@health.ucsd.edu

SUPPLEMENTAL INFORMATION

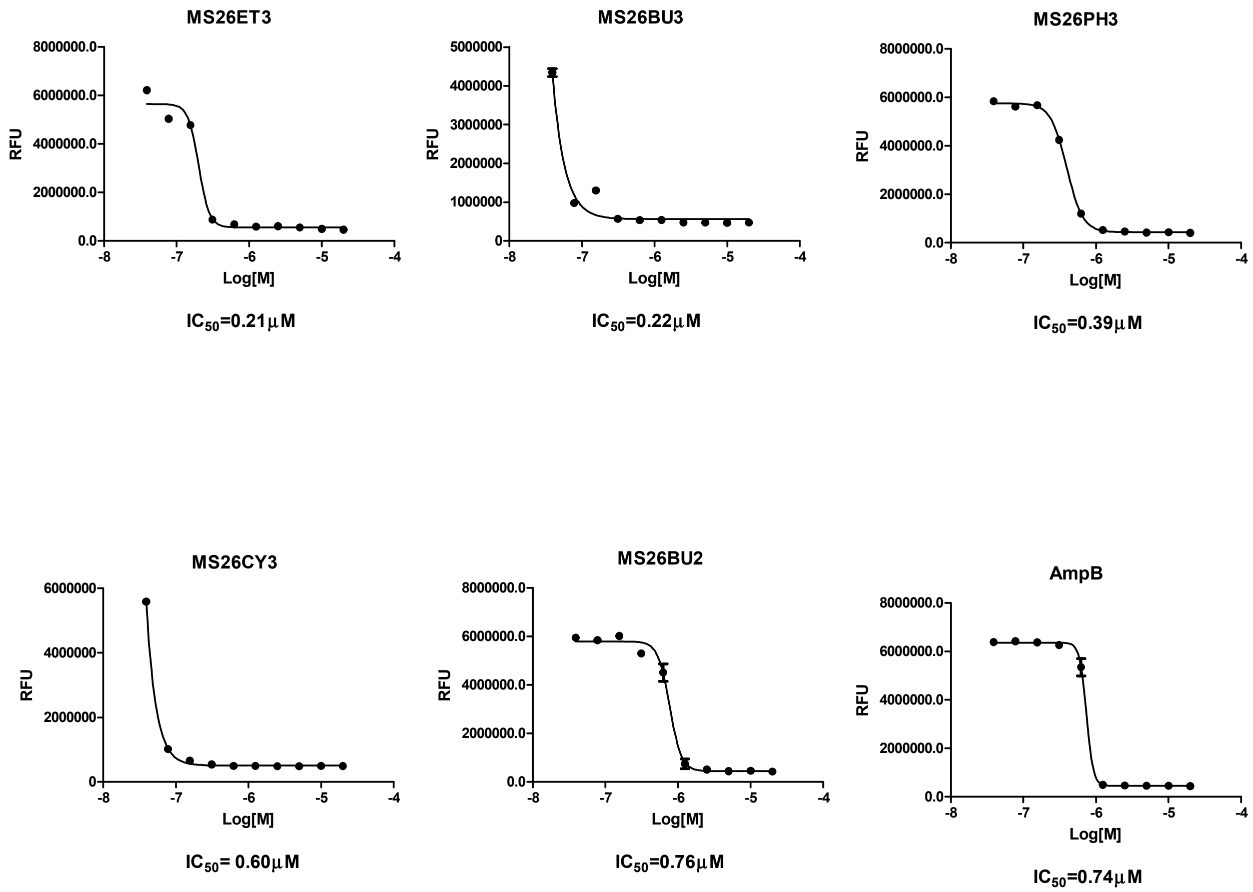

Figure S1. Dose response curves of the potent organotin (IV) derivatives and amphotericin B (control drug) against the promastigotes of *L. donovani.* The RFU measurement corresponds to parasite viability (the higher RFU value, the more viable parasites).

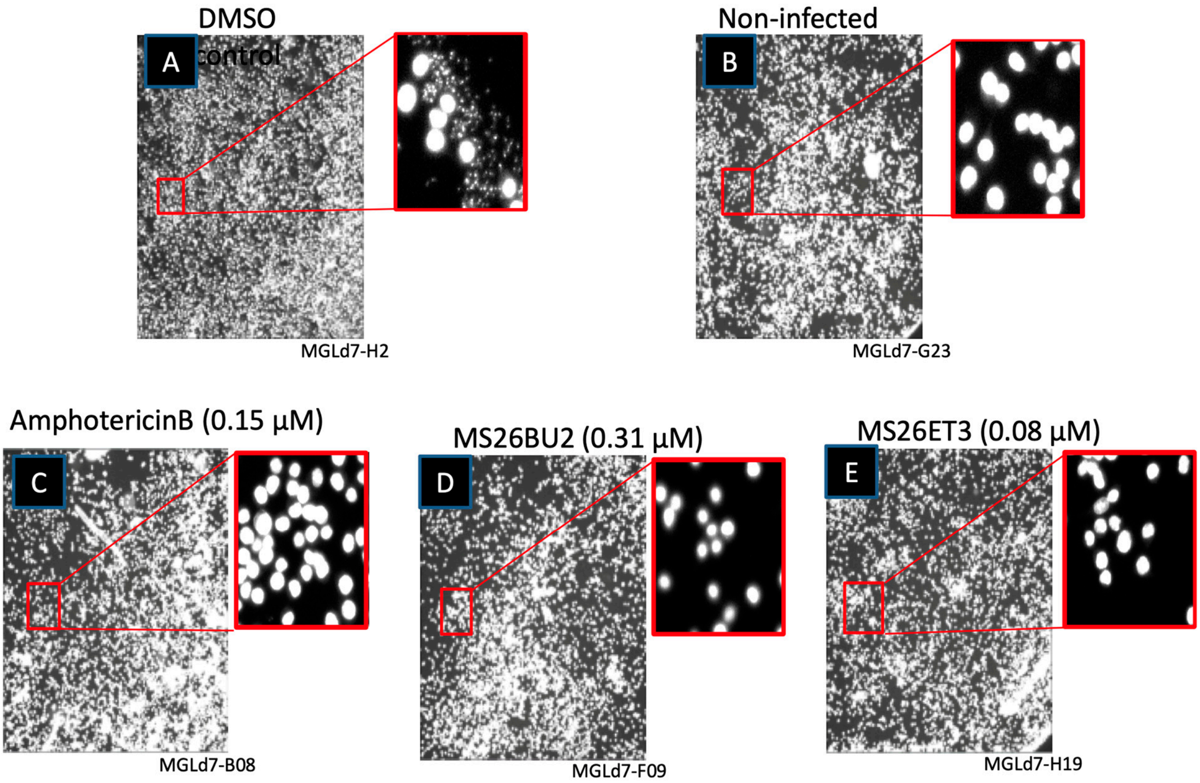

Figure S2: Inhibition of intracellular *L. donovani* amastigotes by organotin (IV) derivatives. ImageXpress Micro XLS automated microscope was used at 10x magnification for imaging host cells and parasites stained with DAPI. **A.** DMSO vehicle-treated B10R macrophages cell lines infected with *L. donovani*. **B.** Non-infected B10R cells. **C.** Amphotericin B (0.15 μM) treated B10R cells infected with *L. donovani*. **D.** MS26BU2 (0.31μM) treated B10R cells infected with *L. donovani*. **E.** MS26ET3 (0.08 μM) treated B10R cells infected with *L. donovani* intracellular amastigotes.

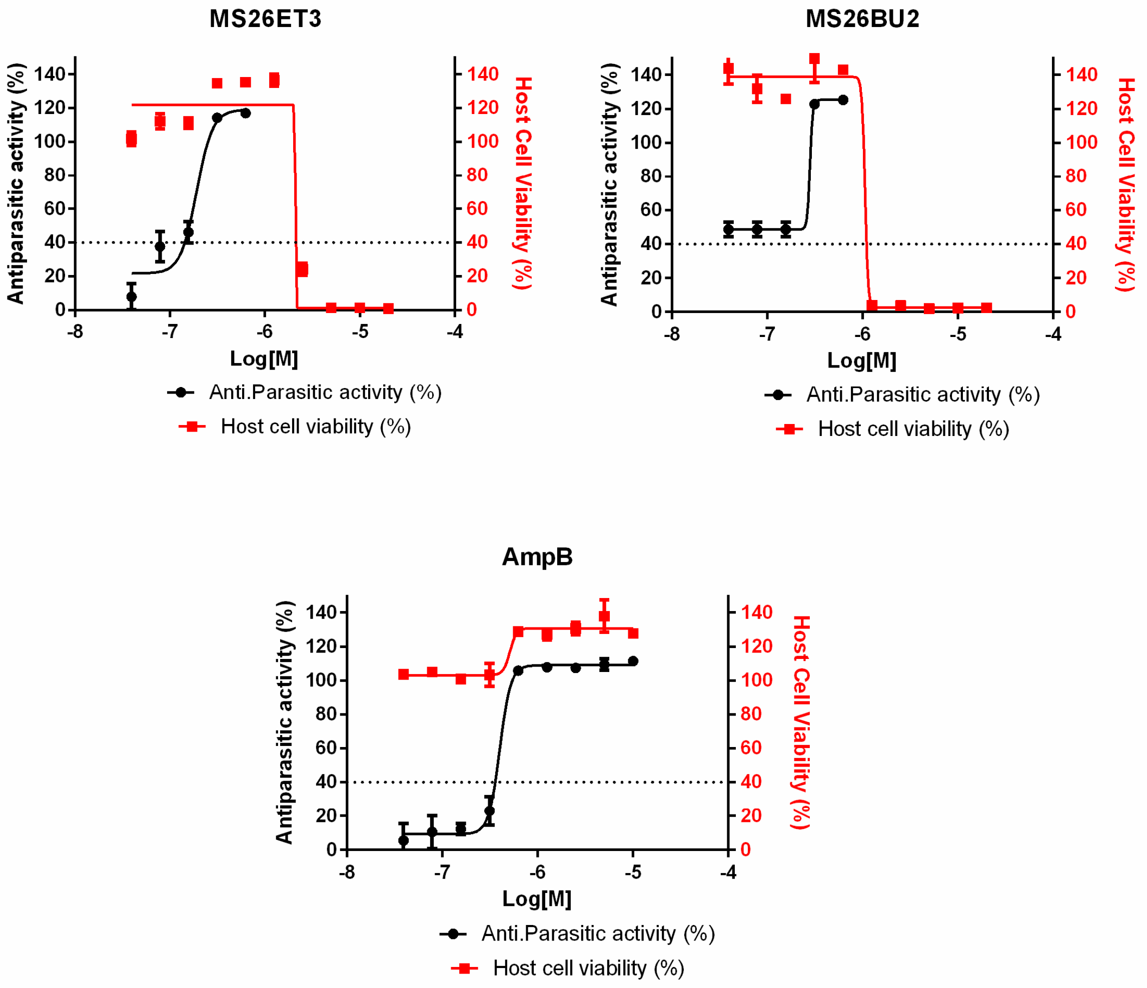

Figure S3. Dose-response curves of the potent organotin compounds and amphotericin B (reference drug) against intracellular amastigotes of *L. donovani*. X-axis represents the various concentrations in Log[M], the left Y-axis (black) represents the percentage (%) of antiparasitic activity in the black dose-response curves while the right Y-axis (red) represents the host cell viability illustrated by the red dose-response curves.
